# Design and Gamma-Ray Attenuation Features of New Concrete Materials for Low- and Moderate-Photons Energy Protection Applications

**DOI:** 10.3390/ma15144947

**Published:** 2022-07-15

**Authors:** Dalal A. Aloraini, M. Y. Hanfi, M. I. Sayyed, K. A. Naseer, Aljawhara H. Almuqrin, P. Tamayo, O. L. Tashlykov, K. A. Mahmoud

**Affiliations:** 1 Department of Physics, College of Science, Princess Nourah Bint Abdulrahman University, P.O. Box 84428, Riyadh 11671, Saudi Arabia; daalorainy@pnu.edu.sa (D.A.A.); ahalmoqren@pnu.edu.sa (A.H.A.); 2 Ural Federal University, 19 Mira St., 620002 Ekaterinburg, Russia; mokhamed.khanfi@urfu.ru (M.Y.H.); otashlykov@list.ru (O.L.T.); 3 Research Sector, Nuclear Materials Authority, El-Maadi, Cairo P.O. Box 530, Egypt; 4 Department of Physics, Faculty of Science, Isra University, Amman 11622, Jordan; 5 Department of Physics, Farook College (Autonomous), Kozhikode 673 632, India; 6 LADICIM (Laboratory of Materials Science and Engineering), University of Cantabria, E.T.S. de Ingenieros de Caminos, Canales y Puertos, Av./Los Castros 44, 39005 Santander, Spain; pablo.tamayo@unican.es

**Keywords:** concrete, NaI (Tl), linear attenuation coefficient, shielding features

## Abstract

We aimed, in this investigation, to prepare novel concretes which can be used in gamma-ray shielding applications. The experimental approach was performed using a NaI (Tl) detector to measure the concrete’s shielding features for different energies, ranging from 0.081 MeV to 1.408 MeV. The density of the fabricated concretes decreased with increasing W/C ratio, where the density decreased by 2.680 g/cm^3^, 2.614 g/cm^3^, and 2.564 g/cm^3^ for concretes A, B, and C, respectively, with increases in the W/C ratio of 0.4, 0.6, and 0.8, respectively. When the energy was elevated between 0.08 MeV and 1.408 MeV, the highest values were attained for concrete A, with values ranging between 0.451 cm^−1^ and 0.179 cm^−1^. The lowest half-value layer (Δ_0.5_) values were achieved for concrete C, where the Δ_0.5_ values varied between 1.53 cm and 3.86 cm between 0.08 MeV and 1.408 MeV. The highest Δ_0.5_ values were achieved for concrete A, where the Δ_0.5_ varied between 1.77 cm and 4.67 cm between 0.08 MeV and 1.408 MeV. According to this investigation, concrete A has the highest promise in radiation shielding purposes because it has the most desirable properties of the concretes studied.

## 1. Introduction

Gamma-ray is electromagnetic radiation that is frequently utilized in diverse fields, including nuclear energy, nuclear medicine, agriculture, industry, dental centers, and many other fields. It is known that gamma photons are massless and chargeless, travel at the speed of light, and have high penetration ability; therefore, the shielding of the gamma ray is very difficult. Thus, the development of novel materials, with sufficient attenuation ability, is an important challenge facing workers in the field of radiation shielding materials. When nuclear engineers plan to fabricate shielding materials, many factors must be taken into account, in order to obtain materials with interesting attenuation performance. These factors include the density, the thickness of the medium type, and energy of the radiation [1,2,3,4,5,6]. 

Heavyweight concrete is concrete that possesses a density higher than 2.6 g/cm^3^ and it is a good choice as a protective material in some applications. It is also used for the storage of high-level nuclear waste. The low cost and simple preparation method are the main reasons that encourage civil and nuclear engineers to focus on developing certain kinds of concrete with different aggregates as shielding materials against low and medium γ-ray energy (E_γ_). Moreover, some types of concrete are composed of a mixture of light and heavy nuclei, so can be applied not only to shield the γ-photons, but also to shield the neutrons [7,8].

Several previous works have shown that when the density of the medium is increased, the attenuation performance of this medium is improved. One common way to increase the density is by adding heavy materials. Accordingly, a few years ago, researchers were interested in enhancing the attenuation performance of concrete by adding heavy aggregates, such as hematite [9], magnetite [10], cementitious composites [11], and barite [12].

Tamayo et al. prepared a high-density concrete, utilizing siderurgical aggregates, and experimentally studied the radiation attenuation factors for the prepared concretes. They also investigated the impact of the W/C ratio on the attenuation parameters [13]. 

Abo-El-Enein et al. prepared ordinary Portland cement pastes with hematite nanoparticles and ZnO nanoparticles and studied the influence of these nanoparticles on the thermal resistance and mechanical properties features. According to their results, the addition of 0.05% ZnO nanoparticles had a negative impact on the radiation protection efficiency of the prepared samples [14].

Roslan et al. fabricated high-density concretes with ferro-boron aggregates and studied their radiation attenuation ability. They found that the density was enhanced with the addition of ferro-boron, and that the concrete mixed with ferro-boron has superior shielding properties to the control specimens. They concluded that the addition of ferro-boron causes an enhancement in photon attenuation capabilities [15].

Liu et al. fabricated barite concretes using waste materials and studied the lead leaching, drying shrinkage, and mechanical properties. They used MCNP simulations to examine the gamma-ray shielding features. They demonstrated a reduction in the photon shielding features with the addition of funnel glass [16].

In this investigation, we aim to fabricate new concretes that may be utilized in the radiation shielding field, using a NaI (Tl) detector to predict the concretes’ attenuation properties between 0.081 and 1.408 MeV. 

## 2. Materials and Methods

### 2.1. Sampling and Preparation

**Figure 1:**
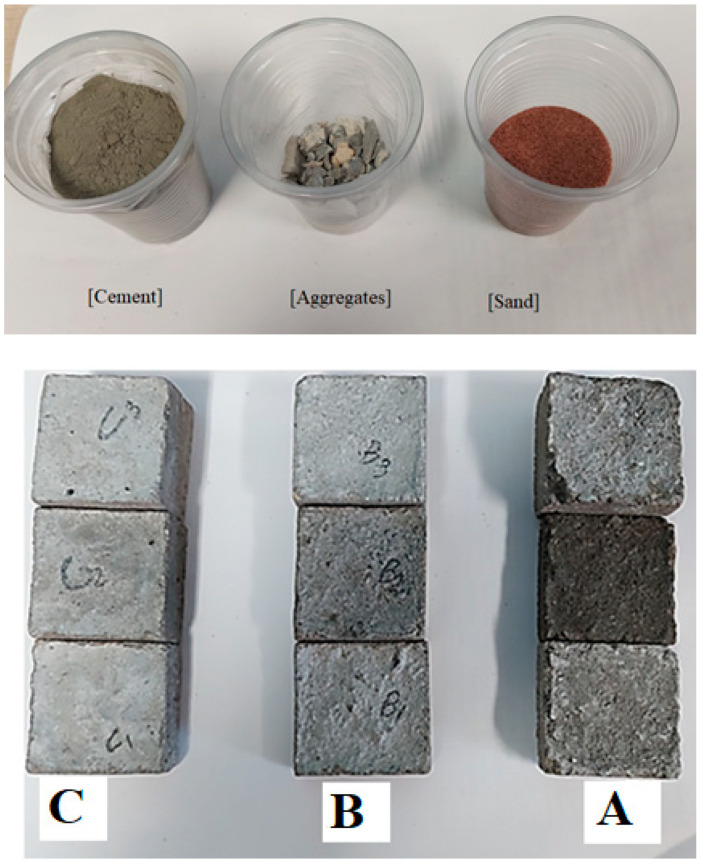
The components used in the concrete fabrication as well as the prepared concrete sample C, B, A with W/C ratio of 0.8, 0.6, and 0.4, respectively.

The concretes were prepared as a mixture of Portland cement, granite (coarse aggregate), and red sand (fine aggregate) with ratios of 1:3:2 by volume. Firstly, the ratio of each component used in the preparation was determined using an electric balance with an accuracy of ±0.01 g. After that, the weighted ratios of granite, cement, and sand were manually mixed well for 15 min. Thereafter, the chosen water ratio was added to the mixture and the effect of water/cement (W/C) on the porosity, density, and radiation shielding parameters was studied. The W/C ratios selected in the study were 0.4, 0.6, and 0.8. The mixture was then molded in the steel cubic molds with dimensions of 7 cm (length) × 7 cm (width) × various thicknesses and the prepared concretes, with different thicknesses, were left to dry for 15 days. Finally, the density was determined using the MH-300A densimeter, with an accuracy of 0.001 g/cm^3^ (Table 1). The materials used in the concrete fabrication, as well as the fabricated concrete samples, are presented in Figure 1. The porosity of the concrete samples was determined by comparing the pore volume (*V_p_*) to the bulk volume (*V_b_*). The *V_p_* was obtained by comparing the difference in mass between the saturated and dry specimen. The porosity was determined according to Equation (1), with resulting values listed in Table 1.

(1) $$Porosity\;\left(\Phi \right)=\frac{V_{p}}{V_{b}}\;$$

### 2.2. Radiation Shielding Capacity

**Figure 2:**
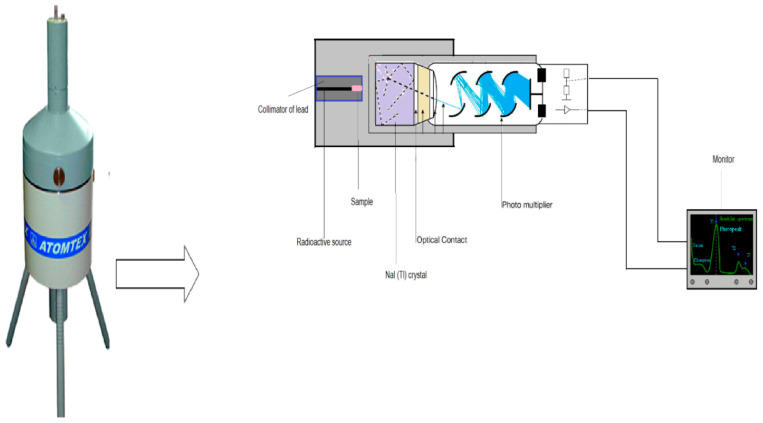
The experimental setup for the attenuation factors measurements.

The linear attenuation coefficient (*µ*) describes the capacity of the shielding material to tolerate crossing photons, and it is the most important protecting feature (see Figure 2). This research aimed to use experimental measurement in order to evaluate the *µ* values for the prepared concretes. The experiment involved using γ-ray sources: ^133^Ba, ^22^Na, ^152^Eu, ^137^Cs, and ^60^Co. The *µ* values were evaluated using a narrow beam transmission method, via a NaI (Tl) detector. The detector recorded incident and transmission intensities, both with and without the constructed concrete (*I* and *I_o_*). The produced concrete samples were all the same thickness. We plotted the relation between (*I*/*I_o_*) and the sample thickness, and from the slope of the obtained curve determined the *µ* value for each concrete sample [17,18,19].

The experimental *µ* is based on the relationship between gamma radiation intensity (incident (*I_o_*) to transmitted (*I*)) and sample thickness (*x*, cm). It is the most important operator for predicting a shield’s propensity to attenuate γ-radiation. The calculation for the linear attenuation coefficient (*µ*) is shown in Equation (2) [20].

(2) $$\mu \left({\mathrm{cm}}^{-1}\right)=\frac{1}{x}ln\;\left(\frac{I_{o}}{I}\right)\;$$

The half-value layer (Δ_0.5_) can be calculated from *µ* values, namely [21].

(3) $$\Delta_{0.5}=\frac{ln\;\left(2\right)\;}{\;\mu \;\left({\mathrm{cm}}^{-1}\right)}\;$$

The transmission factor (*TF*, %) is a parameter that can be used to calculate the rate of γ- ray passing through a known thickness of concrete and is shown in Equation (4) [22]. Moreover, the radiation protection efficiency is defined in Equation (5):

(4) $$TF\;\left(\%\right)=\frac{I}{I_{o}}\;$$

(5) $$\mathrm{RPE}=(1-\frac{I}{I_{o}})\;\;\times \;100\%$$

## 3. Results and Discussion

**Figure 3:**
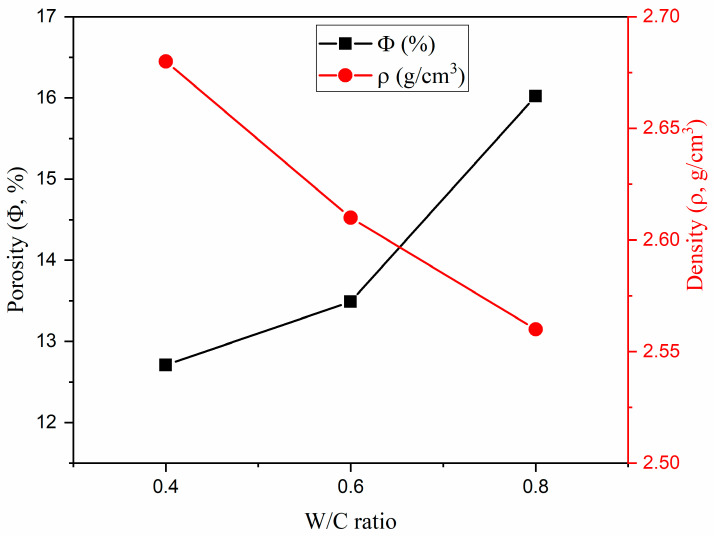
Effect of the W/C ratio on the density and porosity of the fabricated concretes.

The W/C ratio has a great impact on the structural properties *V_p_*, *V_b_*, Φ, and the density of the fabricated concretes. Raising the W/C ratio from 0.4 to 0.8 increased the *V_p_* values from 6.68 to 8.83% and was accompanied by a slight increase in *V_b_* values from 53.75 to 54.90%. The increase in pore volume combined with the increase in the bulk volume caused an increase in the porosity of the concrete from 12.4 to 16.1%, as shown in Figure 3, which also indicates that the increase in the Φ values of the concrete samples had a negative effect on the concrete density (ρ, g/cm^3^), where the ρ values decreased slightly, from 2.68 to 2.56 g/cm^3^.

**Figure 4:**
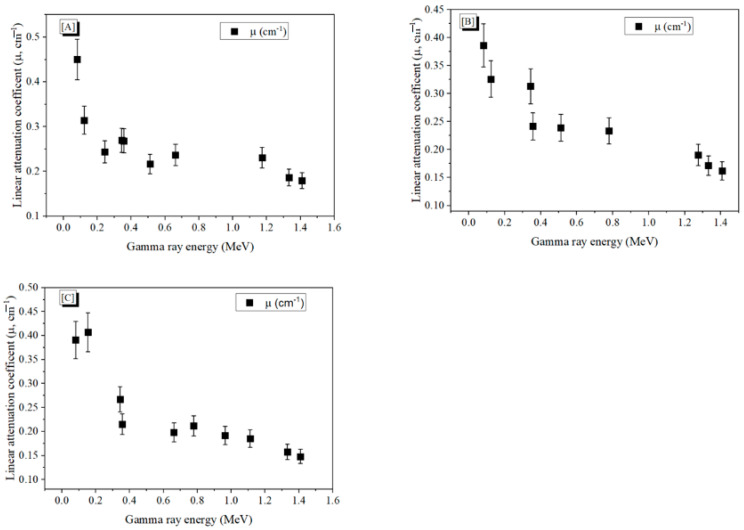
The linear attenuation coefficient versus the incident E_γ_ for concretes coded (**A**), (**B**), and (**C**), respectively.

Radiation shielding factors were measured experimentally, to determine the concretes’ ability to resist the γ-photons. Figure 4 exhibits how the incoming γ-photon energy (E_γ_) from the applied radiation sources affected the *µ* values. Figure 4 displays that the *µ* diminished with the elevation of the incoming energy from the γ-photons. At low energies, the photoelectric effect (PE) is the reason for high *µ* values, and one must remember that the PE cross-section is inversely proportional with the E_γ_ (σ_PE_ α E_γ_^−3.5^) [23,24,25]. With increased elevation of E_γ_ to above 0.1 MeV, the Compton scattering effects (CS) began to change the behavior of the *µ* values, which fell progressively in line with the rising E_γ_ values. These changes in the *µ* behavior were attributed to the proportionality of Cs cross-section with the incoming E_γ_ (σ_com_ α E^−1^). As clarified in Figure 4, concrete A displayed the highest *µ* values among the fabricated concretes, followed by concretes B and C. The *µ* values for all concretes decreased when the energy was increased from 0.081 to 1.408 MeV. The highest *µ* values were achieved for concrete A, where the *µ* varied between 0.451 cm^−1^ and 0.179 cm^−1^ when the energy was raised between 0.08 and 1.408 MeV. The lowest *µ* values were achieved for concrete C, where the *µ* values diminished to between 0.390 cm^−1^ and 0.148 cm^−1^ when the applied E_γ_ was raised between 0.08 and 1.408 MeV, respectively. There were some small anomalies observed in the *µ* values at some discrete gamma-ray energies; thus, the measurements were repeated four times and the uncertainty in measurements was calculated. 

**Figure 5:**
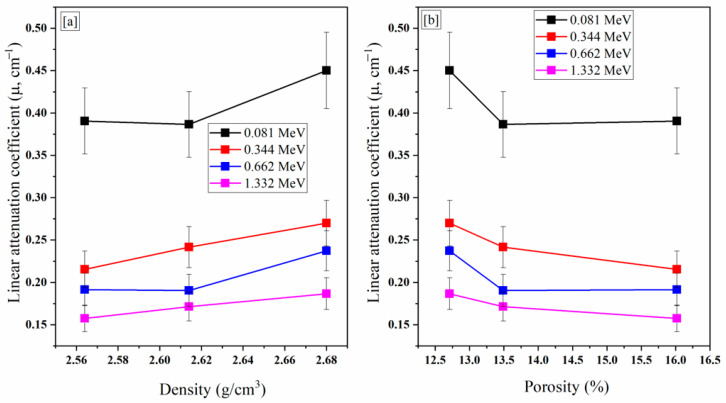
The variation of the *µ* values as a function (**a**) concrete density and (**b**) concrete porosity.

The fabricated concretes’ Φ and ρ were strongly affected by the concretes’ measured *µ* values. Figure 5 A shows a reduction in the measured *µ* values, in the E_γ,_ range from 0.081 to 1.408 MeV, as a result of raising the concretes’ ρ values. Figure 5a also shows that the *µ* values were enhanced, from 0.215 to 0.270 cm^−1^, raising the concretes ρ values from 2.56 to 2.68 g/cm^3^, respectively, at E_γ_ of 0.344 MeV. The same trend was observed for other E_γ_ values, where the *µ* value was raised from 0.157 to 0.186 cm^−1^ at E_γ_ of 1.332 MeV. The present study showed that increasing the W/C ratio in fabricated concrete caused a notable reduction in the concretes’ ρ, which consequently caused a negative effect on the *µ* values and other shielding properties. The ρ values were reduced, ranging between 2.68, and 2.564 g/cm^3^ for concretes A, B, and C, as the W/C ratio was increased to values of 0.4, 0.6, and 0.8. This decrease in the ρ values, with increases in the W/C ratio, may be attributed to the hypothesis that “raising the W/C ratio leads to the formation of air bubbles inside the concrete layers”. These air bubbles were destroyed and formed pore holes after drying the fabricated concretes. Raising the W/C ratio was usually associated with an increase in the number of pore holes created inside the fabricated container, which had a negative effect on gamma-ray shielding.

In order to confirm this hypothesis, the Φ values were studied (see Figure 5b). Figure 5b illustrates the role of the concretes’ Φ values in reducing the measured *µ* values for the fabricated concretes. At E_γ_ of 0.081 MeV, the measured *µ* changed from 0.45 cm^−1^ to 0.39 cm^−1^, due to the increase in the Φ values, from 12.71 to 16.02%, respectively. Similarly, at E_γ_ of 0.662 MeV, the *µ* values decreased from 0.237 to 0.197 cm^−1^ with the rise in Φ values. This decrease in the *µ* ay be attributed to the increase in the pore volume inside the concrete.

**Figure 6:**
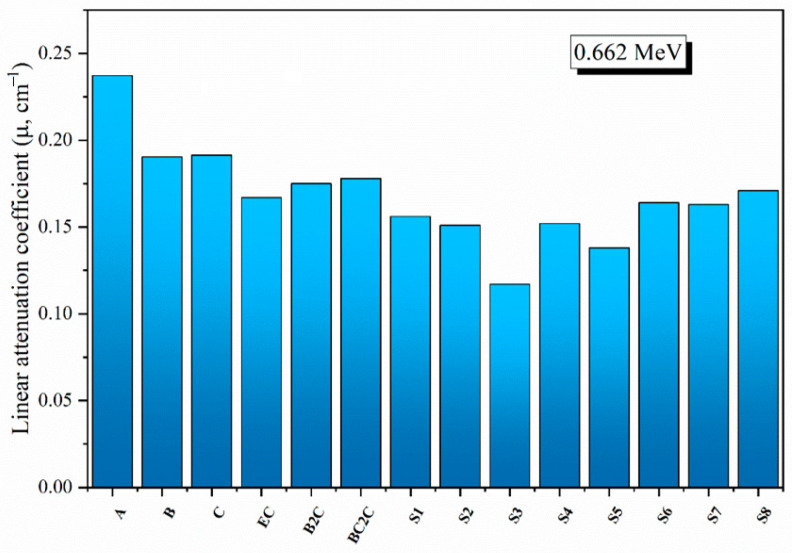
Comparison of the *µ* values for the fabricated concretes and previously published concretes, at E_γ_ = 0.662 MeV.

To validate the shielding ability for the current concretes, a comparison between the measured *µ* values of the fabricated concrete and those for the other previously reported concretes was performed (see Figure 6). The selected samples (previously reported in the literature) contain heavy natural minerals, such as barite, witherite [26], and sepiolite [27]. The comparison illustrates that concrete A, which was fabricated in the present study, has the highest *µ* values, reaching 0.237 cm^−1^. Moreover, samples B and C have close *µ* values, at around 0.190 and 0.191 cm^−1^. These mentioned *µ* values for samples B and C are comparable to concretes B2C and BC2C, with *µ* values around 0.175 and 0.178 cm^−1^, respectively. Similarly, the fabricated samples A, B, and C have higher *µ* than S1–S8 samples, which contain sepiolite minerals. 

**Figure 7:**
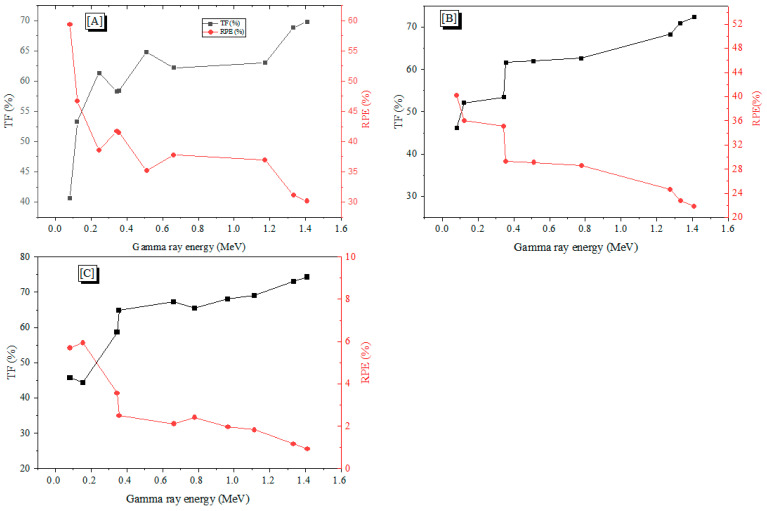
The TF and RPE values as a function of the incoming γ-ray energy (E_γ_) for the three fabricated concrete series (**A**), (**B**), and (**C**) with W/C ratios 0.4, 0.6, and 0.8.

The TF predicts the photon transmittance through the concretes. Figure 7a–c show the interdependence of γ-photon transmission and E_γ_ values. As presented in the Figure, the TF values were relatively small at low E_γ_ where the TF took values of 40%, 46%, and 45% for A, B, and C concretes, respectively, at E_γ_ of 0.081 MeV. This is because the E_γ_ in this energy interval was not enough to escape from the concrete thickness, and the photon energy was almost consumed during the interaction (PE interaction). For all fabricated samples, the TF values were highly increased when raising the incoming E_γ_. This increase was attributed to the predominant CS interaction inside the concrete samples, where CS interaction allowed the photons to accumulate inside the sample, throughout the experiment, consuming only a small part of the incoming E_γ_ during the interaction. The TF values for concrete A (for example), increased from 40% to 70 % when E_γ_ varied between 0.081 and 1.408 MeV (Figure 7).

The calculated RPE values for the fabricated concrete are also shown in Figure 7. The RPE decreased as the E_γ_ increased, allowing high-energy photons to pass through the concrete with ease. For example, the RPE of concrete A dropped from 70% at 0.081 MeV to 40% at 1.408 MeV. Similarly, the RPE values for concrete B and C decreased in intervals, which varied from 54 to 27% (for concrete B) and from 55 to 25% (for concrete C), when E_γ_ varied between 0.081 MeV and 1.408 MeV.

**Figure 8:**
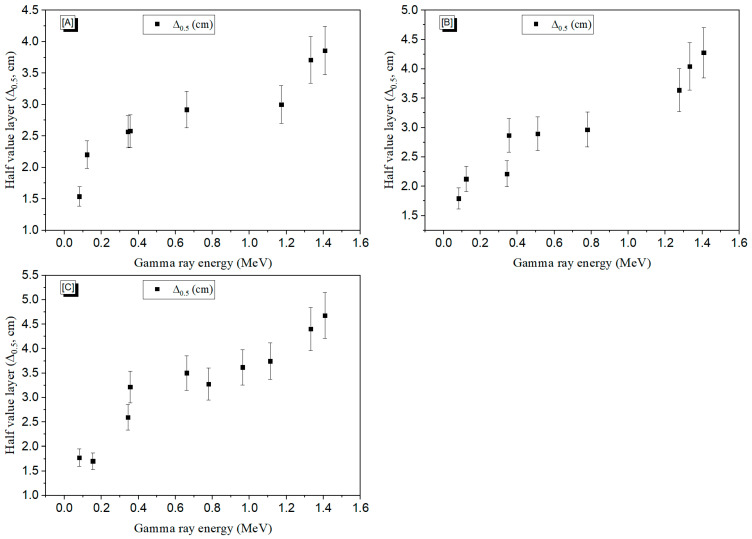
The variation of HVL (Δ_0.5_) with the incoming gamma energy for concretes (**A**), (**B**), and (**C**) with W/C ratios of 0.4, 0.6, and 0.8.

The Δ_0.5_ can describe the fabricated concretes’ ability to reduce the number of transmitted γ- photons. As shown in Figure 8, the Δ_0.5_ values fluctuated with the rise in incoming E_γ_, allowing the required thickness of the concrete composition for radiation shielding to be identified. Conversely, the Δ_0.5_ of the fabricated concretes was increased by raising the E_γ_ and the W/C ratio. The lowest Δ_0.5_ values were achieved for concrete A, where the Δ_0.5_ values varied between 1.53 cm and 3.86 cm when the E_γ_ was raised from 0.08 to 1.408 MeV. The increase in the Δ_0.5_ for the fabricated concrete samples can be attributed to the same reasons discussed in the *µ* section.

## 4. Conclusions

The present work studied the W/C ratio effect on the radiation shielding properties of concretes. Therefore, three samples of concrete were fabricated, using a mixture of Portland cement, red sand, and granite aggregates, with ratios of 1:2:3. The W/C ratios were adjusted to be 0.4, 0.6, and 0.8. The measurements for the fabricated concretes’ ρ values showed that the ρ values slightly decreased from 2.68 to 2.56 g/cm^3^, when the W/C ratio was raised from 0.4 to 0.8. Moreover, the porosity calculation showed that the Φ values inside the fabricated concrete increased when the W/C ratio was raised. Furthermore, an experimental approach was performed in order to assess the radiation protection features of the fabricated concretes with the E_γ_ range extended from 0.081 to 1.408 MeV. The highest *µ* values observed for concrete A decreased from 0.451 cm^−1^ to 0.180 cm^−1^ at values of 0.081 and 1.408 MeV, respectively. The examinations were confirmed using the computed other shielding parameters, such as TF, RPE, and Δ_0.5_. The TF of the fabricated concretes was observed to be significantly diminished with the raising of the incoming E_γ_. Conversely, the RPE values were reduced due to the increase in the incoming E_γ_. As can be seen, the fabricated concrete A had the highest values of TF (70%, at 1.408 MeV) and RPE (60% at 0.081 MeV). Based on the measurements of RPE and Δ_0.5_, it was established that fabricated concretes are preferred in many radiation shielding applications.

## Figures and Tables

**Table 1:** The concrete constituent component ratios, density, and porosity of the fabricated concrete samples.

Concrete Series	The Grain Composition (%)			W/C Ratio	Pore Volume (%)	Bulk Volume (%)	Density (g/cm^3^)	Porosity (%)
	Cement	Granite	Red Sand					
A	16.7	50	33.3	0.4	6.68	53.76	2.68	12.4
B	16.7	50	33.3	0.6	7.39	54.85	2.61	13.5
C	16.7	50	33.3	0.8	8.83	54.90	2.56	16.1

## Data Availability

Data is contained within the article.
